# Adoptive NK cell therapy in AML: progress and challenges

**DOI:** 10.1007/s10238-025-01559-5

**Published:** 2025-01-17

**Authors:** Mona Rady, Maha Mostafa, Gabriel Dida, Fatima Sabet, Khaled Abou-Aisha, Carsten Watzl

**Affiliations:** 1 Microbiology, Immunology and Biotechnology Department, Faculty of Pharmacy and Biotechnology, German University in Cairo (GUC), New Cairo, Egypt; 2 University of South Wales, Pontypridd, Wales UK; 3 Faculty of Biotechnology, German International University, New Administrative Capital, Egypt; 4 Department of Health Systems Management and Public Health, Technical University of Kenya, Nairobi, Kenya; 5 Immunology Department, Leibniz Research Center for Working Environment and Human Factors at TU Dortmund (IfADo), Dortmund, Germany

**Keywords:** Acute myeloid leukemia (AML), Adoptive cell therapy (ACT), Hematopoietic stem cell transplantation (HSCT), Graft versus host disease (GVHD), Graft versus leukemia (GVL), NK cells

## Abstract

Adoptive cell therapy (ACT) using natural killer (NK) cells has emerged as a promising therapeutic strategy for acute myeloid leukemia (AML), addressing challenges such as chemotherapy resistance and high relapse rates. Over the years, clinical trials and studies have explored various sources of NK cells, including ex vivo expanded NK cell lines, CAR-NK cells, peripheral blood-derived NK cells, and umbilical cord blood-derived NK cells. These therapies have demonstrated varying degrees of therapeutic efficacy, ranging from transient anti-leukemia activity to sustained remission in select patient groups. Toxicity profiles have generally shown favorable safety outcomes, with minimal incidence of severe adverse effects such as cytokine release syndrome (CRS) or graft-versus-host disease (GVHD). However, persistent challenges remain, including limited NK cell persistence, relapse, and heterogeneity in patient responses. This review provides a comprehensive analysis of clinical outcomes and toxicity profiles provided from clinical trials, clinical studies and case reports conducted in the last 15 years to judge on the efficacy, safety and applicability of using NK cells for ACT of AML. Our review highlights the significant potential of NK cell-based therapies for AML, while addressing the technical and biological challenges that must be overcome to enhance their efficacy and safety.

## Background

AML is one type of hematological malignancy characterized by uncontrolled proliferation of immature cells of myeloid cell origin, so-called myeloblasts, formed during the process of hematopoiesis [20]. This uncontrolled proliferation of myeloblasts along with evasion of apoptosis lead to the accumulation of those myeloblasts in bone marrow disrupting the process of normal hematopoiesis. The immature myeloblasts fail to develop to normal white blood cells of myeloid origin such as dendritic cells, monocytes, macrophages and granulocytes. It is believed that the abnormal myeloblasts originate from leukemic stem cells and that’s why they exhibit heterogeneous cellular properties in different patients [59]. Therefore, the fact that AML is a heterogeneous and diverse disease urged scientists and clinicians to explore novel personalized therapeutic modalities. Most therapies for AML rely on conventional chemotherapy. Chemotherapeutic approaches can fail sometimes due to resistance of cancer cells and cannot protect against relapse or achieve sustained remission for AML patients [43]. In fact, one of the major challenges in treatment of AML is the high relapse rate after complete remission [51]. Overall, the prognosis of AML is still considered unsatisfactory, in spite of the extensive studies in understanding the biology of AML [11, 19, 20].

AML is a disease of the immune system which can be treated by hematopoietic stem cell transplantation (HSCT) [51], restoring the ability of the immune system to kill those leukemic myeloblasts. In fact, for high-risk AML, allogenic HSCT remains the most powerful therapeutic intervention [13]. The capability of donor immune cells to recognize and kill leukemic cells is termed graft versus leukemia (GVL) effect [59]. However, HSCT, besides not applicable in some patients, can attack recipient’s self-tissues such as skin, liver and gut. Thus, HCST can be associated with graft versus host disease (GVHD), hence associated with morbidity and mortality related to transplantation [43]. Additionally, leukemic myeloblasts can be targeted and eliminated by cytotoxic cells of the immune system such as T-cells and natural killer (NK) cells, making AML an ideal candidate disease that can be targeted through harnessing the power of the immune system in specifically eliminating cancer cells, an approach termed adoptive cell therapy (ACT) of cancer [3, 31]. ACT of AML is a novel immunotherapeutic approach that depends on the direct infusion of immune cells to cancer patients. ACT of cancer aims at eliminating neoplastic cells that acquired special mechanisms to evade the immune systems of cancer patients [59]. One approach of adoptive cell therapy relies on using NK cells.

In fact, Aversa et.al showed that HLA haplotype-mismatched ('haploidentical') HCST can control AML relapse without causing GVHD, a major limitation of HSCT [4]. This highlighted beneficial role of NK cell alloreactivity in the GVL [54] setting the stage for the use of NK cells for ACT in cancer.

## NK cells

**Fig. 1:**
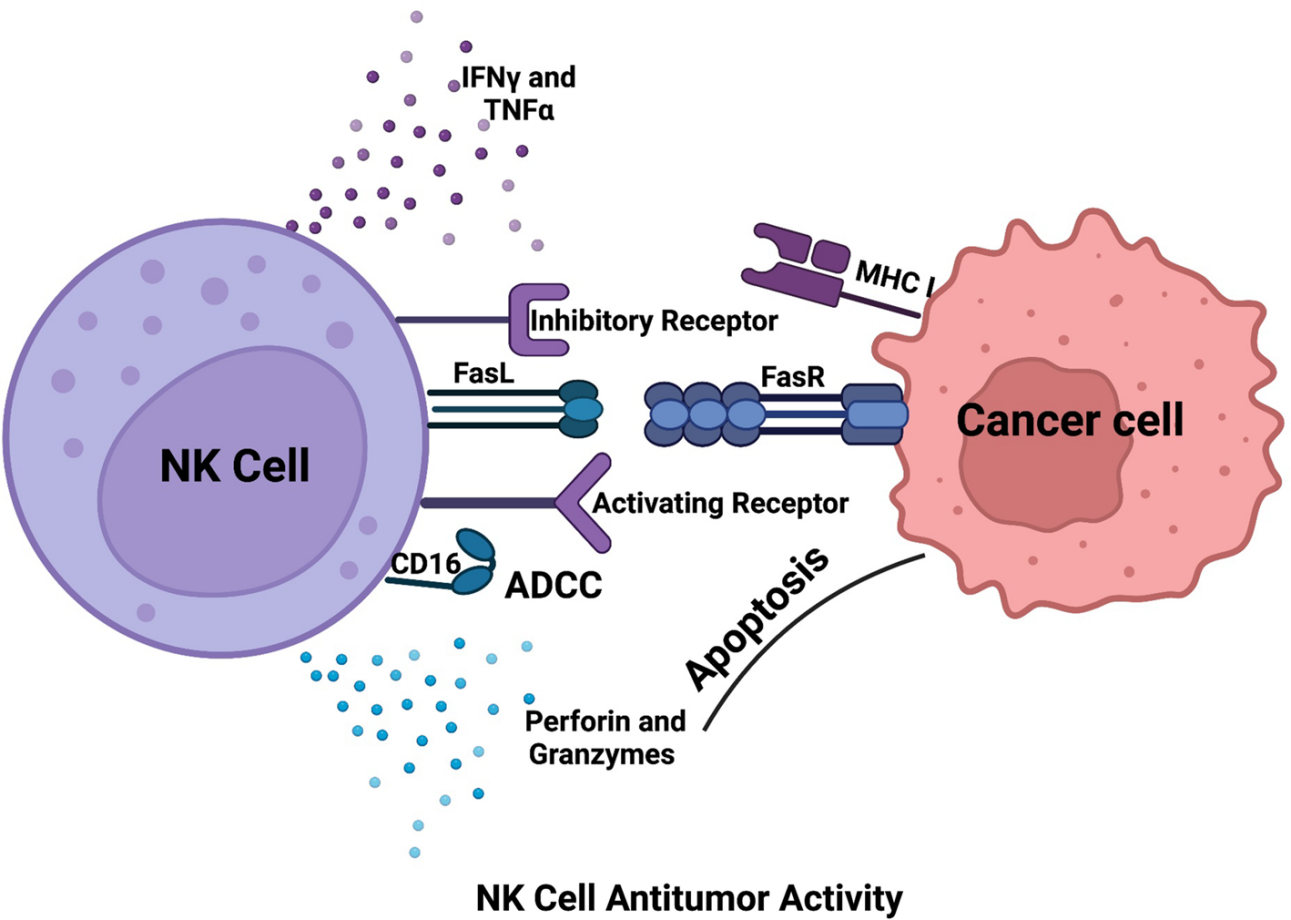
NK cell antitumor activity. NK cells have several functions where it secretes inflammatory cytokines as IFNγ and TNFα. It can perform apoptosis either by the secretion of granzymes and perforin or through FasL-FasR interaction. It has activating receptors that can bind to activating molecules expressed on tumor cells and activate NK cell function. It can also kill cancer cells by the help of IgG through ADCC. ADCC, antibody dependent cell mediated cytotoxicity; CD16, cluster of differentiation 16; FasL, Fas ligand; FasR, Fas receptor; MHC I, major histocompatibility complex class I; IFNγ, interferon gamma; TNFα, tumor necrosis factor alpha. Created in https://BioRender.com

NK cells are group I innate lymphoid cells (ILCs 1) that are large and granular with a cell surface phenotype of positive expression of CD56 (CD56^+^) and absence of CD3 (CD3^−^). They make up about 2–18% of the total lymphocytes available in the peripheral blood (PB) and they “naturally” function to induce cytotoxicity without prior exposure to an antigen, unlike T and B lymphocytes, which undergo extensive genomic re-arrangements during their development [48]. NK cells are considered the first line of defense against cancer and infection [58]. They help in early fighting of viral infections and cancer with the help of a battery of activating receptors such as CD16, C-type lectin-binding receptors,NKp80 (a marker of maturity expressed by functionally mature NK cells present in the secondary lymphoid tissue (SLT and PB), NKG2D and CD94/NKG2C heterodimer, natural cytotoxicity receptors (NCR; NKp46, NKp44, NKp30), the nectin binding adhesion molecule, CD226/DNAM1 (DNAX Accessory molecule 1), activating killer cell immunoglobulin-like receptor (KAR), and the SLAM-related receptors (SRR) NTB-A, 2B4, and CRACC [35, 44]. Figure 1 shows a summary of NK cell antitumor activity. NK cells in AML patients, however, showed reduced cytotoxic potential which suggests that restoring NK cell functions in AML patients is essential to avoid relapse and ensure complete remission after chemotherapy [59].

The attractive feature of NK cells is that they can be transferred relatively safely to patients without the risk of developing GVHD which is a common complication of adoptive cell therapy based on other cell types such as T-cells. Previous attempts of using T-cell as an adoptive cell therapy for AML did not prove to be promising due to the high incidence of associated GVHD and toxicities [51]. This is in spite of the fact that leukemic blasts express antigens that can be attacked by T-cells [59].

## Mechanisms of evasion of NK cells by AML

**Fig. 2:**
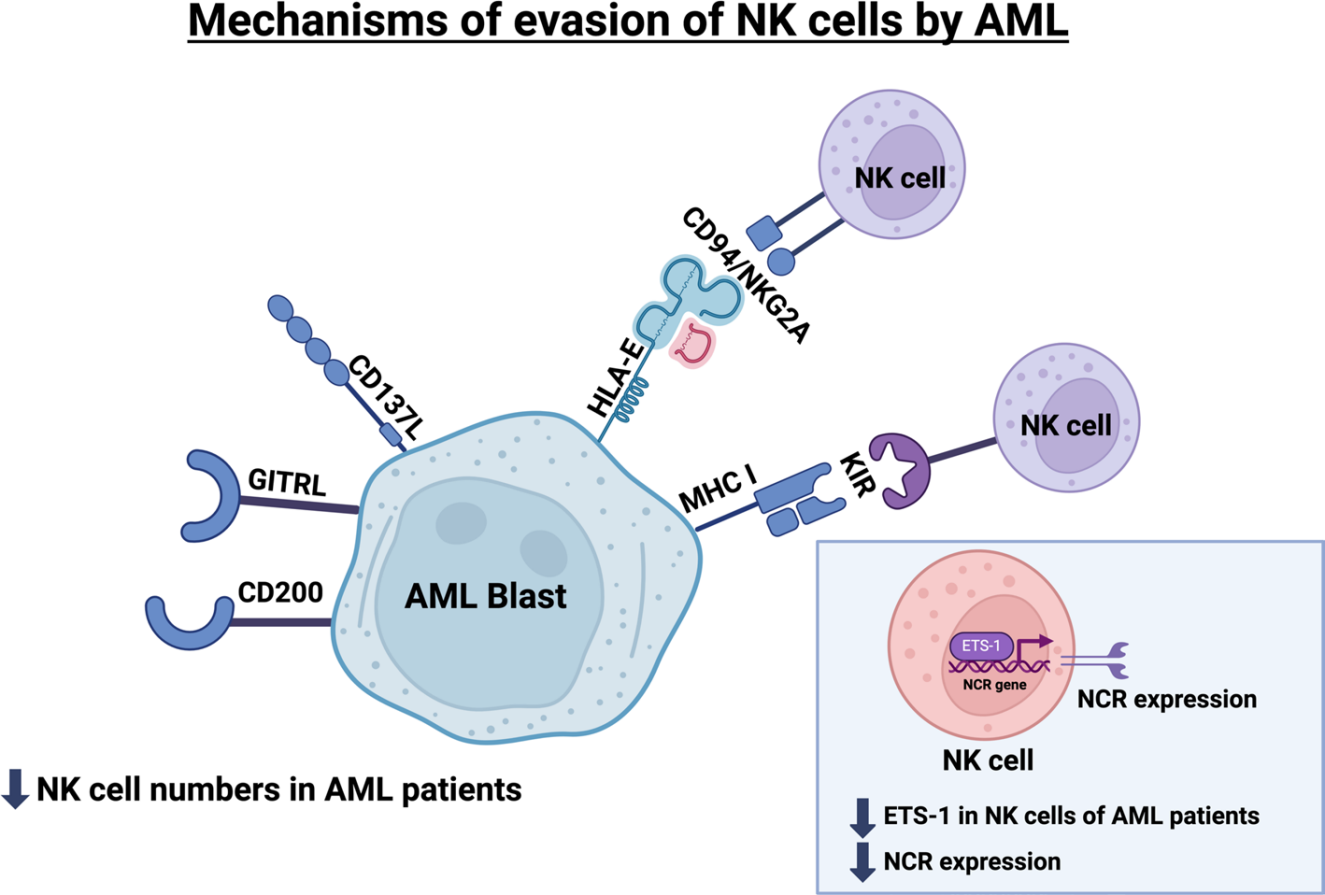
Mechanisms of evasion of NK cells by AML.AML cells can evade the NK cell activity through several mechanisms. This can be either due to defective NK cells with regards to their function or number of infiltrating NK cells. AML blasts also express immunosuppressive factors like GITRL and CD137L. These ligands bind to receptors that belong TNFR family which cause defective killing and decrease IFN-γ production by NK cells. CD200 is another immunosuppressive factor produced by AML blasts that caused lower expression of natural cytotoxicity receptors (NCRs) on NK cells and IFN-γ production. Lower levels of ETS-1 in NK cells of AML patients were also proven. ETS-1 is a transcription factor that binds to part of the promoter region of the NCR gene. Also, higher expression of inhibitory receptor CD94/NKG2A, that bind to HLA-E on AML cells inhibit NK cell function. AML, acute myeloid leukemia; CD200, cluster of differentiation 200; CD94, cluster of differentiation 94; CD137L, CD137 ligand; ETS-1, ETS Proto-Oncogene 1 Transcription Factor; GITRL, glucocorticoid-induced tumor necrosis factor receptor related protein ligand; KIR, kill inhibitory receptor; NK, natural killer cell; MHC I, major histocompatibility complex class I. Created in https://BioRender.com

AML involves immune evasion of NK cells by several mechanisms. This can be either due to defective NK cells with regards to their function or number of infiltrating NK cells [45], leukemic blasts’ downregulation of ligands that bind NK cell activating receptors, production of immunosuppressive factors by leukemic cells or by their interaction with other immune cells [32]. Another mechanism of immune evasion of NK cells is the shedding of activating ligands by leukemic blasts in serum. Thus, chronic engagement of NK cell activating receptors by ligands shed in serum decreases the expression of the NK activating receptor resulting in reduced NK cell cytotoxic potential against leukemic cells [32]. AML blasts also expresses immunosuppressive factors like glucocorticoid-induced tumor necrosis factor receptor related protein ligand (GITRL) and CD137L. These ligands bind to receptors that belong to the tumor necrosis factor receptor (TNFR) family which cause defective killing and decrease IFN- γ production by NK cells [6]. CD200 is another immunosuppressive factor produced by AML blasts that caused lower expression of natural cytotoxicity receptors (NCRs) on NK cells and IFN- γ production [15]. One study showed that AML patients with lower NCRs expression had poor prognosis and lower 5-year survival rate [23]. Lower levels of ETS-1 in NK cells of AML patients were also proven. ETS-1 is a transcription factor that binds to part of the promoter region of the NCR gene and plays an important role in regulating the three NCRs [32, 56]. Immune escape in AML can also occur due to higher expression of inhibitory receptor CD94/NKG2A, that bind to HLA-E on AML cells, and KIR specifically KIR2DL2 on NK cells [26]. This results in decreased NK cell mediated cytotoxicity leading to immune escape by AML. AML cells also have the ability to overcome apoptosis by altering their apoptosis machinery or the expression of death receptors [32]. One study was conducted and showed that patients who administered decitabine (DAC), a hypomethylating agent, had higher levels of the activating NKG2D ligands that activate the activating receptor NKG2D which led to enhanced NK cell mediated cytotoxicity. Moreover, DAC enhanced antibody dependent cellular cytotoxicity (ADCC) which means better binding to the activating receptor CD16 compared to control subjects [53]. DAC also caused increased activating receptor DNAM-1 levels, inflammatory cytokines, perforins and TRAIL [12, 32]. Interaction of AML or NK cells with other immune cells can also cause immune escape. Low cytotoxic NK cells in AML are unable to get rid of immature dendritic cells (DCs). This can lead to a defect in the T cell tolerization [52]. AML patients also showed increased levels of regulatory T cells (Treg) compared to healthy subjects [52]. The increased Treg levels affected NK cell function and decrease levels of responder T cells directly or indirectly through soluble factors like transforming growth factor β1 (TGF-β1) and IL-10 [49]. A study conducted on mice proved that low cytotoxic NK cells affected DCs therefore reduced stimulation of T cells [22]. [56] conducted a study and showed that NK cell percentage in healthy donors (HD) is 2.4% ± 2.19 higher compared to AML patients. The proliferation of the NK cells and NCR expression in HD was also higher compared to AML patients. Defective NK cell function or decreased NK cell number increased the chances of developing cancer, relapse after treatment and death especially in hematological malignancies [33]. Figure 2 summarizes the mechanisms of evasion of NK cells by AML.

## Research question and search strategy

Taken together, this raises the question; what is the evidence that NK cell-based adoptive cell therapy of AML is effective, safe or applicable? Here we review the clinical studies and trials and case reports published in the last 15 years tackling the use of NK cells as an adoptive cell-based therapy for AML to judge on its clinical efficacy, safety and applicability.

The PUBMED database was searched for the term “adoptive cell therapy” as a general term that includes NK cells as one of the cells used for adoptive cell therapy of cancer. The search results were filtered to case reports, clinical trials and clinical studies and to the species, humans, and published in the English language in the last 15 years. One hundred and ninety articles were carefully skimmed for titles to include only articles tackling the use of NK cells as an adoptive cell therapy for AML. From this search results, the articles obtained included all immune cell types used for adoptive cell therapy of other types of diseases including all types of malignancies and infectious diseases. Therefore, the articles tackling immune cell types other than NK cells for adoptive cell therapy such as T-cells, cytokine-induced killer cells (CIK), tumor infiltrating lymphocytes (TIL) or dendritic cells were excluded. Also, articles tacking the potential use of NK cells for battling other types of hematological or solid malignancies or infectious diseases were excluded. In addition, the PUBMED was searched without any filters for the terms “chimeric antigen receptors and NK cells and AML” and “adoptive cell therapy and AML.” Seventeen articles in total matched the inclusion criteria and were included in this literature review.

## Findings

### NK cell sources for adoptive cell therapy for AML

The availability of a suitable and feasible NK cell source is critical to the usability of NK cells for adoptive transfer to AML patients. Different studies used different NK cell sources that were tested pre-clinically for their effectiveness to kill leukemic cells in vitro. The NK-92 cell line expanded ex vivo was used successfully by investigators. The origin of NK-92 cell line is a patient with non-Hodgkin's lymphoma [28]. Using good laboratory practice [10], successfully ex vivo expanded NK-92 cell line in the presence of IL-2, irradiated to inhibit further proliferation, and tested for presence of microorganisms or endotoxin before administration to patients. Also, in another study genetically manipulated NK-92 cell lines were generated to express CD33 chimeric antigen receptor which were shown to have better cytotoxic effect on leukemic cells in vitro [50]. Isolation and expansion of NK cells from peripheral blood donors is also well established in most laboratories. NK cells isolated from eligible donors for haploidentical HSCT were used as an adoptive cell therapy for AML patients who were eligible for HSCT [14, 39, 47] or from haploidentical HLA-matched and killer inhibitory receptor (KIR) mismatch family member donor [30]. A similar strategy was used for patients not eligible for HSCT by isolating NK cells from haploidentical HLA-matched donors [17]. Bachanova et al. relied on administering IL-2 diphteria toxin fusion protein (IL2DT) after adoptive transfer of haploidentical NK cells co-administered with IL-2 for patients with refractory AML [5]. A similar strategy was done to avoid stimulation of Tregs that are produced due to administration of IL-2 along with haploidentical NK cell infusion in refractory AML patients which was to take intravenous (IV) or subcutaneous (SC) recombinant human IL-15 (rhIL-15) [16]. Another study was conducted on elderly AML patients was to increase the number of alloreactive haploidentical KIR-ligand-mismatched NK cells followed by IL-2 to assess the clinical response [18]. Another study produced allogenic ex vivo expanded peripheral NK cells from random healthy unrelated donors. Two infusions from those ex vivo NK cells were administered to 10 patients with refractory/relapsed AML [1].

Fehniger et al. used another strategy to prime NK cells using CTV-1 leukemia cell line lysate CNDO-109 to be called CNDO-109 NK cells that can be used against cell lines that are resistant to NK cells [24].

In a phase I/II clinical trial, elderly patients with MDS/AML, de novo AML and refractory high risk MDS were infused with haploidentical NK cells without IL-2 intake after administering less intense lymphodepleting (LD) regimen [9]. Another phase 2 trial was conducted on children with intermediate risk AML administering low intensity LD regimen followed by infusing highly purified KIR-HLA mismatched NK cells from haploidentical donors and SC IL-2 [38]. Another phase II clinical trial took place in which same donor-memory like (ML) NK cells were infused along with N-803 (IL-15 superagonist) to patients with relapsed/refractory (rel/ref) AML after intake of reduced intensity regimen before HLA-HCT [8]. Bednarski et al., conducted another phase I clinical trial on nine pediatric and young adults (YA) with relapsed AML after HCT by treating them with donor lymphocyte and ML NK cell infusion, from the previous HCT donor, 2 weeks after salvage chemotherapy [7]. Another study produced Allogeneic NK cells from random healthy unrelated donors Peripheral NK cells were expanded and two infusions from those ex vivo NK cells were administered to 10 patients with refractory/relapsed AML [1].

Another source of NK cells is to prepare NK cells from CD34+ umbilical cord blood cells isolated from HLA matched umbilical cord blood [21]. This is done by treating CD34+ cells in vitro with suitable cytokines to favor NK cell differentiation yielding highly pure NK cells in optimum amounts required for NK cell dosing [46].

**Fig. 3:**
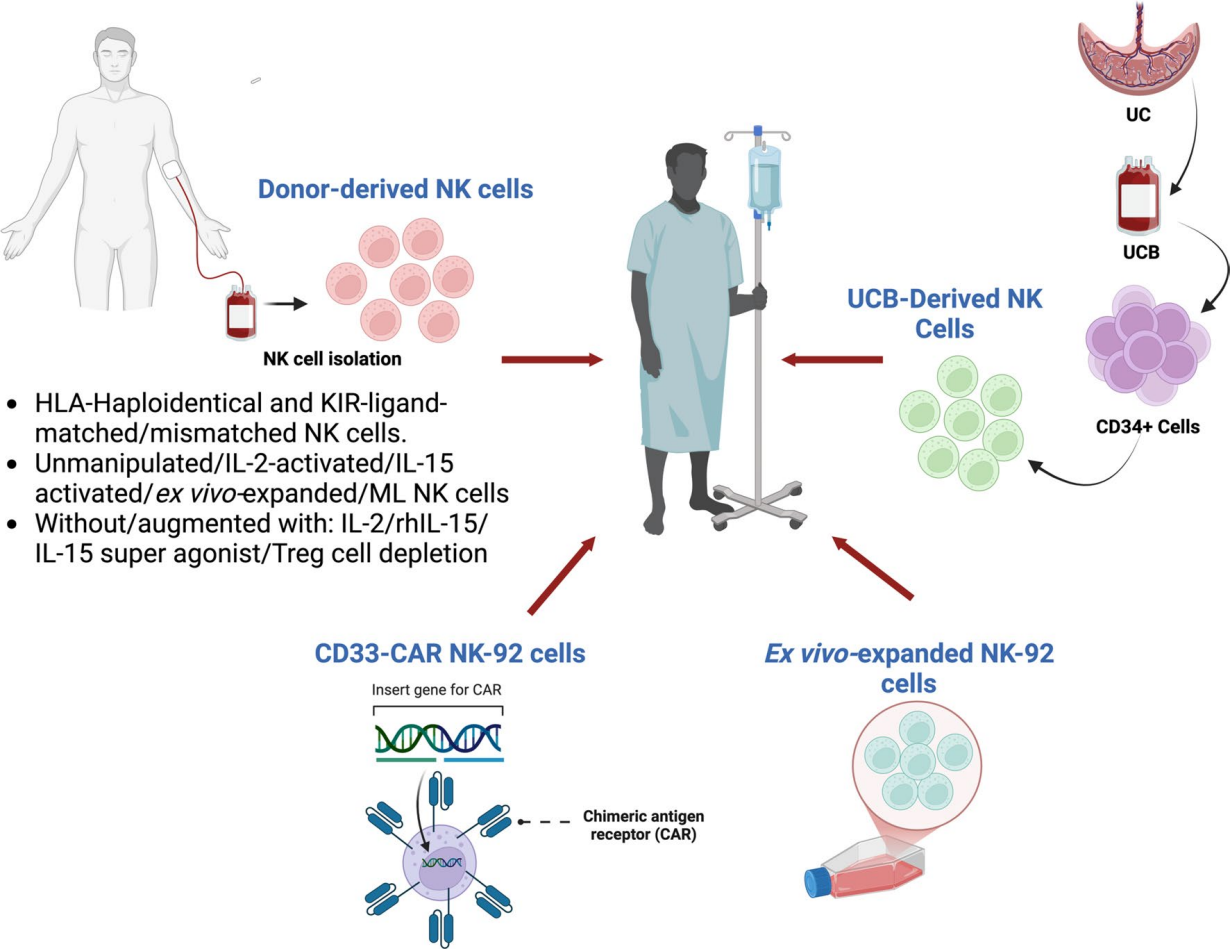
NK cells used for adoptive cell transfer include the use of NK-92 cell line which is expanded ex vivo*.* Also, genetically manipulated NK-92 cell lines were generated to express CD33 chimeric antigen receptor which were shown to have better cytotoxic effect on leukemic cells in vitro. Another source of NK cells is to prepare NK cells from CD34+ umbilical cord blood cells isolated from partially HLA matched umbilical cord blood. Isolation and expansion of NK cells from peripheral blood donors is also used. CD33, cluster of differentiation 33; CD34, cluster of differentiation 34; ML, memory like; NK, natural killer cell; IL, interleukin; rhIL, recombinant human interleukin; UC, umbilical cord; UCB, umbilical cord blood. Created in https://BioRender.com

Figure 3 summarizes the sources of NK cells used for ACT of AML in the studies reviewed. Table 1 summarizes the NK cell dose per infusion and dosing schedules employed in the studies included in this review.

**Table 1:** NK cell dose per infusion and dosing schedules employed

NK cell therapy and NK cell sources	NK cell dose per infusion and dosing schedule	References
Ex vivo expanded NK-92 cell line	Two cell dose levels were used: 1 × 10^9^ cells/m^2^ and 3 × 10^9^ cells/m^2^ One treatment course consisted of two infusions of the same cell dose, each cell infusion administered 24 h apart The maximum number of courses reached 3 courses (6 infusions in total)	Boyiadzis et al. [10]
CD33-CAR-NK-92 cells	Two patients received 3 doses of CD33-CAR NK-92 infusion in December 2017: 3 × 10^8^, 6 × 10^8^, and 1 × 10^9^ cells on days 1, 3, and 5, respectively One patient received 3 doses of CAR NK-92 cell infusion: 1 × 10^9^, 3 × 10^9^, and 5 × 10^9^ cells on days 1, 4, and 7, respectively	Tang et al. [50]
Allogenic ex vivo expanded peripheral NK cells from random healthy unrelated donors	Cohort 1 was initiated with an adoptive transfer dose of 2 × 10^6^ cells/kg for the first injection and 5 × 10^6^ cells/kg for the second infusion (once weekly, two infusions) Cohort 2: Patients received 5 × 10^6^ cells/kg for the first infusion and 10 × 10^6^ cells/kg for the second infusion (once weekly, two infusions)	Ahmadvand et al. [1]
Alloreactive haploidentical KIR ligand-mismatched NK cells followed by IL-2	The median number of infused NK cells was 2.74 × 10^6^/kg	Curti et al. [17]
Post-HSCT NK cell therapy; ex vivo expanded peripheral NK cells isolated from HLA-haploidentical donors	Donor natural killer cell infusion received on days 6, 9, 13, and 20 of HSCT Median NK cell doses were 0.5, 0.5, 1.0, and 2.0 × 10^8^/kg cells, respectively	Choi et al. [14]
Post-HSCT NK cell therapy; peripheral NK cells isolated from haploidentical donors	Sixteen patients received a total of 29 NK cell infusions on days +3, +40 and +100 post-HSCT Median doses (and ranges) of infused NK- and T-cells per product were 1.21 (0.3–3.8) × 10^7^/kg and 0.03 (0.004–0.72) × 10^5^/kg, respectively	Stern et al. [47]
Post-HSCT NK cell therapy; peripheral NK cells from haploidentical related donor and IL-2	A single infusion does of 3 × 10^7^ of donor NK cells/kg was administered on Day 0, which was Day 136 post HSCT	Nguyen et al. [39]
Pre-HSCT NK cell therapy; IL-2-activated peripheral NK cells isolated from HLA-haploidentical donors and KIR-matched or mismatched donors followed by IL-2	Patients were treated in 4 dose levels of the NK cell (1) 10^6^ cells/kg, (2) 5 × 10^6^/kg, (3) 3 × 10^7^/kg, and (4) 3 × 10^7^/kg	Lee et al. [30]
Allogenic haploidentical KIR-ligand-mismatched NK cells followed by IL-2	The median number of infused NK cells/kg was 4.0 × 10^6^ (range 1.29–5.53) and that of infused CD3+ T cells was 0.65 × 10^5^/kg (range 0.1–3.1)	Curti et al. [18]
Allogenic Haploidentical and KIR-matched or mismatched IL-2-activated NK cells augmented with IL-2 and Treg depletion with the IL2DT	Cohort 1: CD3 depletion alone, dose of NK cells/kg infused, 0.96 ± 0.3 × 10^7^ Cohort 2: CD3 depletion followed by CD56 selection, dose of NK cells/kg infused, 0.34 ± 0.05 × 10^7^ Cohort 3: single step CD3/CD19 depletion, dose of NK cells/kg infused, 2.6 ± 1.5 × 10^7^	Bachanova et al. [5]
Haploidentical rhIL-15- activated NK cells followed by SC/IV rhIL-15	SC rhIL-15 group, mean NK-cell dose of 1.2 × 10^7^/kg IV rhIL-15, mean NK-cell dose of 1.9 × 10^7^/kg	Cooley et al. [16]
Allogenic haploidentical KIR–HLA-mismatched donor unmanipulated NK cells and SC IL-2	Median NK cell dose of 12.5 × 10⁶ NK cells/kg (range: 3.6–62.2 × 10⁶ cells/kg)	Nguyen et al. [38]
Cytokine-induced ML NK cells post HLA-haploidentical HSCT followed by 3 weeks of support with N-803 (IL-15 superagonist)	NK cell dose range was 0.5 × 10^6^ to 10 × 10^6^ cells/kg	Berrien-Elliott et al. [8]
Cytokine-induced ML NK cells post HLA-haploidentical HSCT from matched-related, and matched-unrelated donors and post donor lymphocyte infusion	NK cell dose range of 4–15 × 10^6^ cells/kg (maximum dose, 10 × 10^6^ cells per kg) The average dose of NK cells infused was tailored to the patient’s weight and clinical protocol requirements	Bednarski et al. [7]
Ex vivo activated NK cells isolated from related HLA-haploidentical donors using CNDO-109 (IL-15 super agonist)	Patients were treated in escalating doses of trial product at doses of 3 × 10^5^, 1 × 10^6^, or 3 × 10^6^ CNDO-109-NK cells/kg recipient body weight after preparative chemotherapy	Fehniger et al. [24]
IL-2 activated haploidentical NK cells derived from related donors. Two patients received HLA-identical NK cells. KIR-KIR ligand mismatch was present in seven of the sixteen donor-recipient pairs	The median NK-cell dose infused was 6.7 × 10^6^ cells/kg (range, 1.3–17.6 × 10^6^ cells/kg) with T-cell numbers not exceeding 1.2 × 10^5^ cells/kg (range, 0.0–1.2 × 10^5^ cells/kg) as single infusion Two patients received NK cell re-treat	Björklund et al. [9]
Partially HLA-matched with KIR receptor–ligand mismatch and/or KIR B haplotype umbilical cord blood CD34^+^ hematopoietic stem and progenitor-derived NK cells	Escalating HSPC-NK cell dose between 3 and 30 × 10^6^/kg body weight	Dolstra et al. [21]

### Clinical efficacy of NK cell adoptive therapy for AML

Clinical efficacy of adoptive NK cell-based therapy is the determinant to their usability in battling AML. This can be presented in terms of months/years of complete remission, prevention or delaying the incidence of relapse or increase in overall patient survival. Patients with refractory/relapsed AML did not benefit from adoptive NK cell therapy relying on NK-92 cell lines expanded ex vivo [10]. The use of NK-92 cell lines did not result in complete remission in patients with refractory/relapsed AML and only 1 patient showed decreased levels of leukemic blasts following NK-92 cells infusions [10]. Although it is not clear whether the dosing intervals or no concomitant cytokine administration are the results of this treatment failure, it is evident that the use of NK-92 as such is not effective as an adoptive cell therapy for patients with relapsed/refractory AML. The investigators irradiated the NK-92 cells before administration to patient as an essential requirement to stop cellular proliferation, however, there is no evidence that this is the reason for treatment failure as the cytotoxic potential of NK-92 cells against leukemia cells in vitro was not dampened by irradiation. Moreover, genetic manipulation of NK cells to express CD33 chimeric antigen receptors, although showed improved cytotoxic activity against leukemic cells in vitro, did not show any clinical efficacy for AML patients [50]. In their study however, the investigators did notice some reduction in cytotoxic potential of NK cells in vitro after irradiation. Taken together, since irradiation of NK-92 cells is a requirement before administration to patients as their origin is a lymphoma patient, the use of NK-92 cells seems to be of limited clinical applicability if future studies reported reduced cytotoxic potential of NK cells post irradiation.

Similarly, the efficacy of NK cells isolated from haploidentical HLA-matched donors was not consistent among studies. In the study of [17], in high-risk AML patients who were not eligible for HSCT, complete remission was obtained in only 1 of 5 patients with active or progressive AML. In the same study, 2 patients with refractory/resistant AML achieved complete remission which lasted 4 and 9 months [17]. Also in the same study, patients who were treated with NK cell infusions during their complete remission phase were disease free for a period ranging from 18 to 34 months [17].

In the study conducted on 16 patients by Bjorklund et al., 5 had high risk MDS, 3 with de novo AML and 8 with MDS/AML. Out of the 16 patients that administered less intense LD regimen before infusion of haploidentical NK cells, 6 patients showed CR, marrow CR or partial response. In addition, 3 patients showed morphologic leukemia-free state (MLFS) or stable disease (SD). Responding patients had notable reduction of high-risk clones and presence of donor NK cells at day 14 of NK cell infusion [9]. In the phase 2 trial conducted on 21 children with intermediate risk AML, the fraction of KIR-HLA-mismatched, alloreactive NK cells protracted in 17 patients but contracted in 4. Furthermore, 8 patients encountered relapse after NK cell ACT and out of those 8, 3 died of AML. Comparing chemotherapy alone with the combination of chemotherapy and NK cell infusion, NK cell infusion didn’t improve the event-free survival, overall survival nor the relapse rate. In addition, the alloreactive NK cell number didn’t correlate with the overall survival nor the event-free survival during the trial [38]. In the clinical trial of Berrien-Elliott et al., at day 28, 13 out of the 15 patients with rel/ref AML achieved composite CR (CR, Cri or cytogenetic CR (CRc)). Of the other 2, one showed MLFS on day 28 and the other showed partial response on day 28 that converted to Cri on day 44. At day 100, 12 patients were alive of which 4 still showed composite CR. Furthermore, the median OS was 7 months, median event-free survival was 3.2 months and median LFS in 14 of 15 patients with composite CR was 2.2 months. In addition, 4 patients lived more than a year after HLA-HCT of which one showed no relapse and another one relapsed but showed another CR after being infused with donor lymphocytes only. 13 patients stopped treatment due to relapse and 2 patients stopped treatment due to nonrelapse mortality (NRM). Regarding patients with mutations before treatment, 13 patients showed reduced mutations at day 28 and 2 patients showed no mutations showing longest LFS among all patients, longer remission and showed two out of three longest OS in the study. 2 out of 7 patients with mutated TP53 were found clear from them and 8 out of 13 TP53 mutations were not detected at any time after HCT. Moreover, absolute NK cell numbers remained higher than normal for 2 months and specifically ML NK cells were significantly increased above baseline up to day 70 [8]. In the study of Bednarski et al., the donor ML NK cells expanded and persisted for more than 3 months. The study started with 10 patients, one patient died before starting the study and got excluded. Another patient changed from AML to T-cell lymphoblastic leukemia at the beginning of the study but was not evaluable for the efficacy of this treatment regimen. 4 of 8 evaluable patients showed CR at day 28. 2 patients stayed in remission for more than 3 months and at 2 years’ follow-up, OS is 42% and 1 patient remained in remission after ML NK cell therapy only without any additive therapy [7].

Also choosing patients who achieved complete remission after standard chemotherapy confirms that NK cell infusions can prevent relapse and overcome minimal residual disease following chemotherapy.

In the study of [5], 57 refractory AML patients were treated with cyclophosphamide and fludarabine before the NK cell infusion and IL-2 intake. NK cell expansion was 10% in 42 patients, while it was 27% in 15 patients that received IL2DT to deplete the host Treg that can help in improving the efficacy of the haploidentical NK cells transferred. The administration of IL2DT resulted in better complete remission rate at 28 days and better disease-free survival at 6 months [5]. Another study was conducted on 42 refractory AML patients receiving either IV or SC rhIL-15. In phase 1 trial, out of 26 patients, 36% showed in vivo NK cell expansion at day 14 and 32% of them showed complete remission. In a phase 2 trial, 16 patients received SC rhIL-15 and 27% achieved NK cell expansion on day 14 and 40% acquired complete remission [16]. Fehniger et al. [24] also conducted a phase 1 clinical trial on AML patients with first complete remission at high risk for relapse by using CNDO-109-NK cells after lymphodepleting chemotherapy and showed that median relapse-free survival (RFS) at 3 different escalating doses was 105, 156 and 337 days while 2 patients showed RFS of more than 42.5 months [24]. Curti et al. [18] conducted another study on 17 elderly AML patients in first complete remission to test the clinical efficacy of increasing the number of infused alloreactive NK cells after chemotherapy and IL-2 intake. 1 patient died due to bacterial pneumonia and out of the 16 patients, 9 are disease-free whereas the other 7 showed median relapse time of 9 months. Infusing higher number of alloreactive NK cells showed longer DFS [18]. Putting together the data of the aforementioned studies, suggests that NK cell adoptive therapy can be a consolidation therapy to chemotherapy for patients who are not eligible to HSCT or can even be a bridge between chemotherapy and making patients potentially eligible to HSCT.

The clinical outcome of the administration of adoptive NK cell therapy after HSCT was also evaluated. NK cells isolated from the HLA-matched hematopoietic stem cell donors achieved complete remission in 57% of AML patients at 1 month after HCT [14]. However, 74% of AML patient experienced progressive leukemia—3-year cumulative incidence—[14], suggesting that the dosing schedule used which started 6 days after HSCT did not prevent progression of leukemia. Another study evaluated 2 dosing regimens of NK cell infusions post HSCT,one starts 3 days after HSCT and another that starts 40 days after HSCT [47]. Early NK cell infusions did not prevent GVHS or decreased graft rejection. Moreover, 4 of 8 AML patients relapsed after NK cell infusions [47]. In a case report of a patient with refractory AML who relapsed after haploidentical HSCT, received NK cell infusion 136 days after transplant [39]. Although the patient achieved complete remission, he relapsed 80 days after NK cell infusion (216 days after the haploidentical HSCT) [39].

The use of adoptive NK cell therapy was also evaluated before HSCT using NK cells isolated from Haploidentical HLA-matched and KIR-mismatched family member donors other than the HSCT donor. Eleven of 14 high risk AML patients with active leukemic disease state at the time of HSCT achieved complete remission [30] suggesting the benefit of adoptive NK cell therapy prior to HSCT.

In another study, the use of ex vivo expanded peripheral NK cells in 10 patients with refractory/relapsed AML resulted in; one patient was not assessable because of COVID-19 infection and he died while nine patients were evaluated for response assessment after the two NK cell infusions. Six patients showed stable disease (SD) and 3 presented progressive disease (PD). The blast percentage remained stable after the two NK cell infusions within 3 months in 6 patients. Two out of the 6 SD patients remained alive in SD and 3 patients converted to PD at 9 months after infusion of NK cells, and 1 was not assessed due to follow-up loss [1].

The clinical outcome of NK cells derived from haploidentical HLA matched CD34+ cord blood cells was investigated in AML patients during their complete remission after standard chemotherapy protocol and were also not eligible to HSCT [21]. At the time of publication of their study, 4 patients were disease free for 16, 22, 52 and 60 months, 6 patients relapsed after a mean of 364 days. Moreover, patients with minimal residual disease at the time of NK cell infusion showed marked decrease in minimal residual disease signals [21]. An explanation to the relative success of this study is the highly pure NK cells infusions prepared from CD34+ umbilical cord blood cells and the investigators avoided the use of concomitant IL-2 administration. IL-2 is known to increase the levels of T-regulatory cells which impair NK cell functions in vivo [21]. Table 2 summarizes the key therapeutic outcomes and clinical responses from NK cell therapy observed in AML patients.

**Table 2:** Key therapeutic outcomes and clinical response observed from NK cell therapy

NK cell therapy and NK cell sources	Key efficacy outcomes	References
Ex vivo expanded NK-92 cell line	No complete remission for refractory/relapse AML Transient anti-leukemia activity was noted in three of the seven patients	Boyiadzis et al. [10]
CD33-CAR-NK-92 cells	Moderate in vitro cytotoxicity of CAR NK-92 cells against leukemia cell lines Detectable levels of CAR NK-92 cells in the blood post-infusion, albeit with transient therapeutic activity Temporary reduction in tumor burden in one patient, though others showed limited clinical response	Tang et al. [50]
Allogenic ex vivo expanded peripheral NK cells from random healthy unrelated donors	6 of 9 patients showed stable disease; no significant tumor progression in refractory AML 3 of 9 patients presented progressive disease No patient achieved complete remission Among the six patients with SD, two remained in stable condition, while three progressed to PD at 9 months post-infusion	Ahmadvand et al. [1]
Alloreactive haploidentical KIR ligand-mismatched NK cells followed by IL-2	Transient complete remission in 1 of 5 high risk AML patients not eligible for HSCT 2 patients with refractory/resistant AML showed complete remission for 4 and 9 months NK cell infusion during complete remission caused disease free survival from 18 to 34 months	Curti et al. [17]
Post-HSCT NK cell therapy; ex vivo expanded peripheral NK cells isolated from HLA-haploidentical donors	Complete remission in 57% of AML patients at 1 month after HCT 75% experienced leukemia progression (3-year cumulative incidence)	Choi et al. [14]
Post-HSCT NK cell therapy; peripheral NK cells isolated from haploidentical Donors	With a median follow-up of 5.8 years, 4 out of 16 patients were alive at the time of reporting Among the deceased patients, causes included relapse (5 patients), GVHD (3 patients), graft failure (3 patients), and transplant-related neurotoxicity (1 patient) 4 of 8 AML patients relapsed after NK cell infusions	Stern et al. [47]
Post-HSCT NK cell therapy; peripheral NK cells from haploidentical related donor and IL-2	Significant expansion of the infused NK cells was observed, particularly the alloreactive KIR2DL1⁺KIR2DL2/DL3⁻NKG2A⁻ subset, which reached 117 × 10⁶ cells/L by day 14 post-infusion Relapse 80 days after NK cell infusion	Nguyen et al. [39]
Pre-HSCT NK cell therapy; IL-2-activated peripheral NK cells isolated from HLA-haploidentical donors and KIR-matched or mismatched donors followed by IL-2	Among AML patients, several achieved complete remission Relapse-free survival (RFS): median of 102 days Overall survival (OS): median of 233 days GVHD-free/relapse-free survival (GRFS): median of 89 days There was a trend toward improved survival in those who received KIR-mismatched NK cells	Lee et al. [30]
Allogenic haploidentical KIR-ligand-mismatched NK cells followed by IL-2	CR achieved in 69% (11 of 16 evaluable patients) DFS: median follow-up of 22.5 months; 56% remained disease-free Relapse Rate: 44% relapsed within a median of 9 months (range: 3–51 months) High numbers of alloreactive NK cells correlated with reduced relapse	Curti et al. [18]
Allogenic haploidentical and KIR-matched or mismatched IL-2-activated NK cells augmented with IL-2 and Treg depletion with the IL2DT	Remissions at day 28 for 8 of 15 patients (53%), including CR compared to those who did not receive infusion (21%) Disease free survival was notably higher in the IL2DT group (33%) versus the non-IL2DT group (5%) Patients treated with IL2DT exhibited a higher rate of donor NK cell expansion (27%) compared to those who did not receive IL2DT (10%)	Bachanova et al. [5]
Haploidentical rhIL-15-activated NK cells followed by SC/IV rhIL-15	CR or CR with incomplete blood count recovery (CRi) was achieved in 32% of patients receiving intravenous (IV) rhIL-15 and 40% of patients receiving subcutaneous (SC) rhIL-15 SC administration resulted in a median response duration of 278 days compared to 107 days with IV One-year overall survival was 19% for the IV group and 21% for the SC group Both approaches were effective bridges to allogeneic HSCT in some patients	Cooley et al. [16]
Allogenic haploidentical KIR–HLA-mismatched donor unmanipulated NK cells and SC IL-2	Cumulative relapse incidence was similar between treated and control groups (39.3% vs. 35%) EFS at 2 years was 60.7% versus 69.1% for control; overall survival (OS) was 84.2% versus 79.1% Adoptive NK cell therapy did not improve EFS or OS compared to chemotherapy alone Donor NK cell persistence: limited chimerism, with a median peak of 4% donor NK cells in peripheral blood	Nguyen et al. [38]
Cytokine-induced ML NK cells post HLA-haploidentical HSCT followed by 3 weeks of support with N-803 (IL-15 superagonist)	Among the 15 patients treated, 87% achieved a composite complete response by day + 28 post-infusion High response rate correlated with the clearance of high-risk mutations, including TP53 variants ML NK cells persisted for at least 2 months after HCT and were highly functional ex vivo	Berrien-Elliott et al. [8]
Cytokine-induced ML NK cells post HLA-haploidentical HSCT from matched-related, and matched-unrelated donors and post donor lymphocyte infusion	Among 8 evaluable patients, 4 achieved complete remission by day 28 post-infusion Two patients maintained remission for over 3 months, with one patient remaining in remission for more than 2 years Donor-derived ML NK cells expanded and persisted in vivo for over 3 months without the need for exogenous cytokine support	Bednarski et al. [7]
Ex vivo activated NK cells isolated from related HLA-haploidentical donors using CNDO-109 (IL-15 super agonist)	The median relapse-free survival by dose level was 105 days Three durable complete remissions of 32.6 to 47.6+ months were observed Donor NK cell microchimerism was detected on day 7 in 10 of 12 patients, with 3 patients having evidence of donor cells on day 14 or later	Fehniger et al. [24]
IL-2 activated haploidentical NK cells derived from related donors. Two patients received HLA-identical NK cells. KIR-KIR ligand mismatch was present in seven of the sixteen donor-recipient pairs	6 patients (38%) achieved objective responses, including CR, marrow CR, or partial remission Five patients proceeded to allogeneic hematopoietic stem cell transplantation (HSCT), and three patients remained disease-free for over 3 years Responding patients showed a reduction in high-risk mutational clones, suggesting an effective anti-leukemic response	Björklund et al. [9]
Partially HLA-matched with KIR receptor–ligand mismatch and/or KIR B haplotype umbilical cord blood CD34^+^ hematopoietic stem and progenitor-derived NK cells	In patients achieving complete remission after standard chemotherapy and those ineligible for HSCT, 4 patients remained disease-free for 16, 22, 52, and 60 months, while 6 patients relapsed after an average of 364 days Patients with MRD at the time of NK cell infusion experienced a significant decrease in MRD signals	Dolstra et al. [21]

### Safety of adoptive NK cell therapy in AML patients

Using the NK-92 cell lines expanded ex vivo, none of the patient experienced toxicities that would limit the treatment with NK-92 cell infusions [10]. Mild adverse effects were encountered with only 1 patient that were reversed without complications related to the NK-92 cell infusions [10]. Similarly in the clinical trials using the genetically manipulated NK-92 cells expressing the CD33 chimeric antigen receptor did not report serious adverse effects with NK-92 cell infusions [50]. Using haploidentical HLA-matched NK cells after HSCT resulted in marked signs of toxicities represented by weight gain, neurotoxicity, hyperbilirubinemia and fever in a significant number of AML patients [14]. Having those signs of toxicities resolved with no recurrence suggest that HSCT could be the cause of these signs of toxicities. Similarly, administration of NK cell infusions after HSCT resulted in severe GVHD especially in patients who received the highest dose of accumulated T cells in the NK cell preparations [47]. This clearly suggests that the purity of NK cells used for adoptive cell therapy is critical to avoid associated toxicities. In two separate studies, donor NK cell infusions after HSCT did not change GVHD rates commonly observed after HSCT [14, 30]. In high-risk AML patients who are not eligible for HSCT, Haploidentical HLA-matched donor NK cell infusions were considered safe and well tolerated with adverse effects similar to standard chemotherapy regimens [17]. Furthermore, 56% of the patients that received SC rhIL-15 after haploidentical NK cell infusion encountered cytokine release syndrome (CRS) followed by neurological toxicity in 5 out of 9 patients since longer drug exposure occurred. SC intake of rhIL-15 compared to IV intake had slower pharmacokinetic elimination and higher IL-6 levels. Therefore, getting the benefit of SC use of rhIL-15 and decreasing chances of CRS can be through adjusting the dosing regimen based on pharmacokinetics and pharmacodynamics [16]. Using CNDO-109-NK cells in high risk AML patients showed no toxicities even at highest administered dose [24] Regarding increasing the number of alloreactive NK cells infused followed by IL-2 injection, out of the 16 patients, 10 cases had fever for unknown cause, 4 cases showed bacterial infections where 2 developed pneumonia and the other 2 developed sepsis, only 1 patient died due to bacterial pneumonia but others recovered successfully. Mild erythema at injection site also occurred after IL-2 injection but, no GvHD was encountered [18]. The toxicities associated with allogenic haploidentical NK cell therapy augmented with IL-2 and Treg depletion using IL2DT were manageable overall. Notably, there was no infusional toxicity directly linked to IL2DT, no cases of acute cytokine release syndrome, and no patients developed acute graft-versus-host disease (GVHD) or autoimmune disorders. A single Grade 4 infusion-related hypersensitivity reaction was observed but resolved promptly with antihistamines and supportive care. However, later Grade 3–5 toxicities emerged, primarily as a result of prolonged cytopenias and immune suppression, which included infections, fevers, and other complications. These findings highlight the safety of IL2DT during infusion while acknowledging the potential for significant long-term toxicities [5]. Two out of 16 patients which received low intensity LD regimen followed by haploidentical NK cell infusion encountered chills and nausea. Patients 6 and 17 showed CRS associated with hemophagocytic lymphohistiocytosis (HLH). Patient 6 died from AML relapse, HLH and human herpes virus (HHV-6) encephalitis 6 weeks after NK cell infusion. Another well tolerated adverse effect occurred like tachycardia was proven previously to be related to NK cell infusion. Severe adverse effects like infections and cytopenia showed no correlation to NK cell ACT. To conclude, cell infusion was safe with only 2 of 16 patients that showed up with chills and nausea [9]. In the phase 2 trial conducted by Nguyen et al., all 21 patients showed extravasation skin injury caused by cyclophosphamide, 20 developed neutropenia and 5 developed thrombocytopenia. Twenty patients also had absolute neutrophil and platelet count recovery within 45 days. No opportunistic infections, bleeding and GvHD occurred [38]. Regarding a phase II clinical trial conducted by Berrien-Elliott et al., adverse events of ML NK cells and N-803 were minimal and tolerable. No patient had steroid-refractory acute GvHD. 1 patient had grade 1 CRS and patients showed grade 1 or 2 reactions on injection site. Ten patients showed acute GvHD. 2 of 10 evaluable patients had chronic GvHD, one was mild and on skin and the other was moderate involving skin and GIT. These GvHD reactions were compared to ones caused by HCT proving that it was not exaggerated by ML NK cells nor N-803. 2 patients encountered, one from complications of sepsis and other from complications of primary graft failure [8]. Regarding safety of ML NK cell infusion in relapsed AML pediatric patients after allogenic HCT, 2 patients showed low-grade fever due to ML NK cell infusion. Patients also experienced minimal nonhematologic toxicities of grade 3 or 4 and 1 patient had skin GvHD which continued and also showed increased liver enzymes due to liver GvHD. Patients also experienced chemotherapy related toxicities like cytopenia, nausea, vomiting and febrile neutropenia [7].

When using PB-NK cells with 10 AML patients in the study of [1], none of the 10 patients experienced dose-limiting toxicity during the infusion or during the 28 days of the post-infusion observation period. One of these patients died 8 weeks after PB-NK cell infusion because of severe acute respiratory syndrome with a positive test for SARS-COV 2. No grade 2–5 toxicities related to PB-NK cell infusion occurred. Four patients developed grade 1 transient chills, vomiting, bone pain and headaches following each PB-NK cell infusion that didn't need hospitalization. In all cases, they were reversible and responsive to supportive care, intravenous hydration and antipyretics. Taken together, NK cell infusions are considered relatively safe, provided that the preparations are pure and free from residual T-cells during their isolation or expansion ex vivo.

Using NK cells isolated from CD34+ cord blood cells for AML patients who received initial chemotherapy regimen and not eligible to HSCT was not associated with GVHD, severe infections or symptoms of cytokine release syndrome [21]. Only severe cytopenia was observed with patients who heavily received chemotherapy prior to NK cell infusions [21], suggesting that patient selection for adoptive NK cell therapy is critical to avoid associated severe toxicities. Table 3 summarized the toxicities and adverse effects observed with NK cell therapy in AML patients.

**Table 3:** Toxicities and adverse effects observed with NK cell therapy

NK cell therapy and NK cell sources	Toxicities/adverse effects	References
Ex vivo expanded NK-92 cell line	No grade 3 or 4 toxicities related to the NK cell infusions were observed No significant changes in patients' lymphocyte counts, subsets, phenotype, or activity post-infusion Cell dose-dependent effects in plasma cytokine levels were observed without leading to severe adverse events	Boyiadzis et al. [10]
CD33-CAR-NK-92 cells	No substantial dose-limiting toxicities observed up to a dose of 5 × 10⁹ cells per patient One patient experienced grade I CRS, with transient fever and elevated interleukin levels (IL-6 and IL-10) that normalized within 48 h A fever of up to 40 °C occurred in another patient, resolving within two days Despite initial safety, hematological relapse occurred in all three patients within months post-infusion	Tang et al. [50]
Allogenic ex vivo expanded peripheral NK cells from random healthy unrelated donors	No grade 2–5 toxicities related to PB-NK cell infusion were observed Four patients experienced grade 1 transient chills, headaches, vomiting, and bone pain following each infusion; these symptoms did not require hospitalization One patient died due to severe acute respiratory syndrome, though the direct association with NK cell infusion is unclear	Ahmadvand et al. [1]
Alloreactive haploidentical KIR ligand-mismatched NK cells followed by IL-2	No NK cell–related toxicity, including GVHD, was observed Side effects similar to standard chemotherapy	Curti et al. [17]
Post-HSCT NK cell therapy; ex vivo expanded peripheral NK cells isolated from HLA-haploidentical donors	During NK cell infusion, 73% of evaluated patients developed fever (> 38.3 °C) Patients experienced a maximum median weight gain of 13% and a maximum median bilirubin level of 6.2 mg/dL The incidences of grade 2 to 4 acute GVHD and chronic GVHD were 28% and 30%, respectively	Choi et al. [14]
Post-HSCT NK cell therapy; peripheral NK cells isolated from haploidentical Donors	Four patients developed acute GVHD of grade II or higher Early NK cell infusions did not prevent GVHD or decreased graft rejection	Stern et al. [47]
Post-HSCT NK cell therapy; peripheral NK cells from haploidentical related donor and IL-2	The NK cell infusion was well-tolerated, with no major adverse effects reported No incidence of GVHD was observed	Nguyen et al. [39]
Pre-HSCT NK cell therapy; IL-2-activated peripheral NK cells isolated from HLA-haploidentical donors and KIR-matched or mismatched donors followed by IL-2	No toxicities were directly attributed to NK cell infusion in AML patients No increase in GVHD was observed in AML patients following NK cell infusion NK cell infusion did not interfere with the engraftment of allogeneic hematopoietic stem cells in AML patients	Lee et al. [30]
Allogenic haploidentical KIR-ligand-mismatched NK cells followed by IL-2	Fever of unknown origin was observed in 10 patients Injection Site Reactions: Mild erythema from IL-2 injections No clinical or laboratory signs of GVHD observed 4 bacterial infections (2 sepsis, 2 pneumonia, including 1 fatal *E. coli* case) 1 death during neutropenia due to *E. coli* pneumonia	Curti et al. [18]
Allogenic haploidentical and KIR-matched or mismatched IL-2-activated NK cells augmented with IL-2 and Treg depletion with the IL2DT	The infusion of NK cells, both with and without IL2DT, was generally well tolerated Mild CRS was observed in some patients, characterized by low-grade fever and manageable symptoms No patients developed acute GVHD or autoimmune disorders 1 grade 4 infusion-related hypersensitivity reaction, which promptly resolved with antihistamines and supportive care Grade 3 to 5 toxicities, which included infection or fevers due to prolonged cytopenias and immune suppression	Bachanova et al. [5]
Haploidentical rhIL-15-activated NK cells followed by SC/IV rhIL-15	CRS was observed in 56% (mild to severe) with rhIL-15 SC administration and none with IV Neurotoxicity was observed in in 33% with rhIL-15 SC administration and none with IV Injection site reactions was observed in 63% rhIL-15 SC administration and none with IV Common mild-to-moderate adverse effects were observed in both rhIL-15 IV and SC administration Diffuse alveolar hemorrhage, prolonged marrow aplasia was observed with rhIL-15 IV administration and CRS-related fatalities, severe neurotoxicity, intracranial hemorrhage with rhIL-15 SC administration	Cooley et al. [16]
Allogenic haploidentical KIR–HLA-mismatched donor unmanipulated NK cells and SC IL-2	Hematologic toxicity: grade ≥ 3 neutropenia (95%) and thrombocytopenia (24%) Infections: no opportunistic infections or GVHD observed Other toxicities: 1 case of nonhematologic Grade 3 adverse event (cyclophosphamide-related skin injury) Injection site reactions: mild erythema from IL-2 injections Mortality: 1 relapse-related death; no therapy-associated fatalities	Nguyen et al. [38]
Cytokine-induced ML NK cells post HLA-haploidentical HSCT followed by 3 weeks of support with N-803 (IL-15 superagonist)	Infusion of donor-derived ML NK cells was well tolerated The therapy did not exacerbate or trigger GVHD in patients Grade 1 CRS occurred in 2 patients	Berrien-Elliott et al. [8]
Cytokine-induced ML NK cells post HLA-haploidentical HSCT from matched-related, and matched-unrelated donors and post donor lymphocyte infusion	Treatment was well tolerated, with no significant toxicities directly attributed to the ML NK cell infusion No exacerbation or initiation of GVHD after ML NK cell infusion, even in patients with a history of GVHD.	Bednarski et al. [7]
Ex vivo activated NK cells isolated from related HLA-haploidentical donors using CNDO-109 (IL-15 super agonist)	All 12 patients experienced treatment-associated adverse effects, most commonly fatigue (50%) and febrile neutropenia, neutropenia, and thrombocytopenia (each 42%) No dose-limiting toxicities were observed at the highest tested dose No evidence of graft-versus-host disease (GvHD) was reported	Fehniger et al. [24]
IL-2 activated haploidentical NK cells derived from related donors. Two patients received HLA-identical NK cells. KIR-KIR ligand mismatch was present in seven of the sixteen donor-recipient pairs	The NK-cell infusions were well tolerated, with most adverse events being transient and mild-to-moderate Grade 3–4 toxicities, including chills and nausea, observed In 2 out of 16 patients, two patients experienced cytokine release syndrome (grade 3 and 5), with one fatality attributed to hemophagocytic lymphohistiocytosis (HLH) combined with HHV-6 encephalitis and AML relapse Responding patients displayed less pronounced activation of CD8^+^ T cells and lower levels of inflammatory cytokines following NK-cell infusion	Björklund et al. [9]
Partially HLA-matched with KIR receptor–ligand mismatch and/or KIR B haplotype umbilical cord blood CD34^+^ hematopoietic stem and progenitor-derived NK cells	Neither graft-versus-host disease nor toxicity was observed Prolonged cytopenia in patients who heavily received chemotherapy prior to NK cell infusion	Dolstra et al. [21]

## Limitations and challenges of ACT using NK cells in AML

Despite their advantages, several limitations have hindered the widespread clinical success of NK cell-based therapies in AML [42]. The use of autologous NK cells, though widely regarded as safe, has shown limited efficacy in treating AML. This limitation is largely attributed to the expression of inhibitory receptors on autologous NK cells that recognize self-MHC class I molecules presented by cancer cells, resulting in suppression of NK cell activation [40, 55]. Furthermore, autologous NK cells derived from cancer patients are often in an immunosuppressive state with diminished functionality, making it difficult for these cells to exert effective antitumor responses.

Allogeneic NK cell-based therapies, while offering an alternative, present several challenges. Key issues include the requirement for timely ex vivo expansion, insufficient persistence of NK cells in vivo, and suboptimal clinical activation [25, 34]. One major limitation of allogeneic NK cells is the difficulty in generating a sufficient number of functional cells. As a result, recent research has focused on optimizing ex vivo expansion and activation strategies to enhance NK cell activity. Studies have suggested that the systemic administration of cytokines may improve NK cell antitumor responses. However, the use of ex vivo expanded autologous NK cells with IL-15 has been linked to CRS when administered to AML patients [27].

Chimeric antigen receptor (CAR)-NK cell therapy, particularly using the NK-92 cell line, has demonstrated significant promise but faces limitations in terms of persistence and proliferation in vivo due to pre-infusion irradiation [29, 60]. This irradiation compromises the effectiveness of NK-92 cells in ACT [37]. Additionally, cytokine administration is critical for maintaining NK cell persistence in vivo, with IL-15 being preferred over IL-2 due to its reduced likelihood of promoting Treg cell proliferation and its more favorable side effect profile [2, 41]. Moreover, NK-92 cells lack the CD16 receptor, impairing their ability to induce antibody-dependent cellular cytotoxicity (ADCC) [61].

The tumor microenvironment (TME) also presents a significant barrier to CAR-NK cell efficacy, as immunosuppressive cytokines such as TGF-β can reduce the expression of activating receptors and suppress IFN-γ production by NK cells. This suppression severely limits NK cell antitumor activity [57]. CRISPR-Cas9 technology has been employed to address this challenge by knocking out the TGF-β receptor 2 gene (TGFβR2) in primary human NK cells prior to CAR transduction, thereby enhancing NK cell resistance to the immunosuppressive effects of the TME [36].

In the case of NK cells isolated from adult peripheral blood or umbilical cord blood, isolation and in vitro expansion are labor-intensive, with low yields of NK cells. The process typically requires 4 to 10 weeks to generate clinically relevant numbers of NK cells. Another challenge for CAR-NK therapy is the sensitivity of NK cells to the freeze–thaw process, which negatively affects their cytotoxicity and survival rates. However, incubation with IL-2 post-thaw has been shown to restore their functional abilities [36].

Overall, while NK cell-based therapies hold significant potential for treating AML, overcoming these technical and biological challenges is critical for enhancing their clinical efficacy and safety.

## Conclusions, recommendations and future considerations

While promising outcomes are to be expected from NK-cell-based adoptive therapy for AML, the clinical investigations are still in their infancy and suffer a number of limitations. Choosing the eligible candidate patient for this type of therapy is challenging. Moreover, different investigators used different dosing regimens whether in terms of number of NK cells infused or in dosing intervals, with inconsistent results, suggesting that effective dosing and dosing schedules are still unresolved issues. Patients who are not eligible to HSCT seem to be the most suitable candidates—so far—who can benefit from adoptive NK cell therapy following standard chemotherapy regimens [17, 21]. Also, NK cell adoptive therapy is proven to prevent relapse after complete remission achieved from conventional chemotherapeutic regimens [17]. Administration of NK cell infusions did not prove to clinically benefit patients or reduce the incidence of GVHD when administered before HSCT [47]. The use of NK-92 cell lines although can be more feasible did not achieve clinical efficacy [10] or even after genetic manipulation to better target leukemic cells [50]. Further genetic manipulations using chimeric antigen receptors are expected to be a preclinical hot research area to achieve at an NK-92 cell design to prove their preclinical and clinical efficacy. Because the purity of NK cells used is critical to avoid NK cell-associated toxicities, the genetic manipulation of NK-92 cells to express chimeric antigen receptors for better targeting of leukemic cells and simultaneous enhancement of NK cell functions is a promising safe strategy which however, needs extensive preclinical studies.

## Data Availability

No datasets were generated or analysed during the current study.
